# Prevalence and Correlates of Sleep Apnea Among US Male Veterans, 2005–2014

**DOI:** 10.5888/pcd14.160365

**Published:** 2017-06-15

**Authors:** Maylen Jackson, Benjamin J. Becerra, Connie Marmolejo, Robert M. Avina, Nicole Henley, Monideepa B. Becerra

**Affiliations:** 1 Department of Health Science and Human Ecology, California State University, San Bernardino, California; 2 School of Allied Health Professions, Loma Linda University, Loma Linda, California

## Abstract

The objective of this study was to assess the prevalence of and factors associated with sleep apnea among US male veterans. We used data from the 2005–2014 National Survey on Drug Use and Health to conduct survey-weighted descriptive, bivariate, and regression analyses. The prevalence of sleep apnea increased from 3.7% to 8.1% (*P* for trend <.001 for adjusted model) from 2005 through 2014. Increasing severity of psychological distress and unmet mental health care need were associated with increased odds of sleep apnea, as was a diagnosis of asthma. Increased screening of sleep health is critical to improve the health outcomes of veterans.

## Objective

Healthy People 2020 (1) incorporated sleep health as an item on its agenda, calling attention to poor sleep health and its contribution to the national burden of chronic disease. The age-adjusted prevalence of sleep apnea, a chronic condition of disturbed breathing during sleep (2), among US veterans from 2000 to 2010 increased almost 6-fold (3). An evaluation of veterans of Operation Enduring Freedom, Operation Iraqi Freedom, and Operation New Dawn found that 69.2% of 159 veterans screened were at high risk for obstructive sleep apnea (4). Given that sleep apnea is more prevalent among men than among women and that most US veterans are men (5), we sought to assess the prevalence, trends, and risk factors of sleep apnea among US male veterans.

## Methods

We used the 2005–2014 National Survey on Drug Use and Health (NSDUH) public use files (6) to identify US male veteran respondents aged 18 years or older. Sleep apnea, our outcome variable, was defined by using the NSDUH questionnaire (7) that lists numerous health conditions and asks respondents, “Which, if any, of these conditions did a doctor or other medical professional tell you that you had in the past 12 months?” The list includes sleep apnea and asthma. Exposure variables were sociodemographic characteristics (age, race/ethnicity, marital status, federal poverty level, highest education level, and health insurance status) and other self-reported health and behavioral factors: past-year illicit drug use or alcohol dependency, past-year psychological distress, past-year unmet mental health care need, and asthma diagnosed in the past year by a health care professional. Psychological distress, defined by the Kessler 6-scale score, a validated measure of assessing psychological distress (8), was categorized as none (score 0–7), mild to moderate (score 8–12), and serious (score ≥13). Unmet mental health care need was defined by NSDUH as a perceived need for mental health care treatment or counseling that was not received.

We used SAS 9.4 (SAS Institute, Inc) for all statistical analyses; an α of .05 was used to determine significance. Our analytic sample size was 20,631. All data analyses included design-based values to account for the survey weights. First, we used survey-weighted descriptive statistics to evaluate study population characteristics and the prevalence of sleep apnea. We then conducted an analysis of *P* for trend to assess changes in prevalence of sleep apnea during the 10-year study period. We used survey-weighted χ^2 ^tests to evaluate the prevalence of sleep apnea by population characteristic. Finally, we conducted survey-weighted multivariable binary logistic regression to assess the relationship between exposure variables and sleep apnea and accounted for survey year. We assessed relevant interactions to identify effect modifiers. The study was approved by the institutional review board at California State University, San Bernardino.

## Results

The average prevalence of sleep apnea was 5.9% during the study period (Table 1); proportions increased from 3.7% in 2005 to 8.1% in 2014 (*P* for trend <.001 for adjusted model), and the highest prevalence was in 2012 (8.3%) (Figure).

**Table 1:** Selected Sociodemographic, Health, and Behavioral Characteristics of US Male Veteran Respondents Aged ≥18 Years in the National Survey on Drug Use and Health, 2005–2014^a^

Characteristic	Sample Size (Weighted %) (Total n = 20,631)	Average Annual N (Total N = 232,070,104)^b^,
**Age, y**		
18–34	4,523 (7.4)	1,729,477
35–49	5,140 (17.0)	3,944,665
50–64	4,845 (32.4)	7,518,442
≥65	6,123 (43.2)	10,014,426
**Race/ethnicity**		
Hispanic	1,195 (5.2)	1,211,496
Non-Hispanic black	2,020 (9.9)	2,294,377
Non-Hispanic white	16,161 (81.7)	18,948,607
Other	1,255 (3.2)	752,530
**Marital status**		
Currently married	12,581 (69.2)	16,056,914
Not currently married	8,050 (30.8)	7,150,097
**Federal poverty level**		
>200%	15,343 (79.4)	18,431,945
100%–200%	3,785 (15.4)	3,571,030
<100%	1,464 (5.2)	1,198,465
**Education**		
College graduate	4,700 (28.2)	6,550,857
Some college	6,333 (28.1)	6,515,819
High school diploma or less	9,598 (43.7)	10,140,334
**Currently have health insurance coverage**	18,570 (94.0)	21,820,795
**Health and behavioral outcomes**		
Sleep apnea in past year	1,074 (5.9)	1,359,803
Illicit drug or alcohol dependency in past year	1,116 (3.4)	795,654
No psychological distress in past year	16,703 (85.1)	19,756,676
Mild to moderate psychological distress in past year	2,163 (8.8)	2,052,268
Serious psychological distress in past year	1,765 (6.0)	1,398,067
Unmet mental health care need in past year	753 (2.4)	560,992
Asthma in past year	743 (3.9)	888,285

a Data source: Substance Abuse and Mental Health Services Administration (6).

b Sample is weighted to represent an annual population size averaged over 10 years from 2005–2014.

**Figure:**
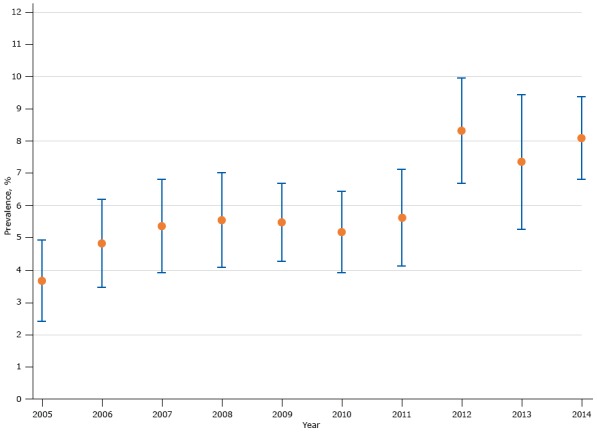
Prevalence of sleep apnea among US male veterans, National Survey on Drug Use and Health, 2005–2014. Error bars indicate 95% confidence intervals. **Table:** 

Year	Prevalence, % (95% Confidence Interval)
2005	3.7 (2.4–5.0)
2006	4.8 (3.4–6.2)
2007	5.4 (3.9–6.8)
2008	5.5 (4.1–7.0)
2009	5.5 (4.2–6.7)
2010	5.2 (3.9–6.5)
2011	5.6 (4.1–7.1)
2012	8.3 (6.7–10.0)
2013	7.4 (5.2–9.5)
2014	8.1 (6.8–9.4)

The greatest percentage of the study population was aged 65 years or older (43.2%), was non-Hispanic white (81.7%), was currently married (69.2%), was living at or above 200% of the federal poverty level (79.4%), and had a high school diploma or less (43.7%). Of the study population, 3.4% reported past-year illicit drug or alcohol dependency. In addition, 8.8% had mild to moderate psychological distress in the past year, 6.0% had serious psychological distress in the past year, and 3.9% had a past-year diagnosis of asthma (Table 1).

We found significant associations between prevalence of sleep apnea and several population characteristics (Table 2). The prevalence of sleep apnea was significantly higher among respondents aged 50 to 64 years (7.9%) than among respondents in other age groups, married respondents (6.6%) than among unmarried respondents, respondents with some college education (7.1%) than among college graduates or those with a high school diploma or less, and those with health insurance coverage (6.2%) than among those without. The prevalence of sleep apnea was 11.4% among respondents reporting past-year serious psychological distress and 5.4% among respondents with no past-year psychological distress. Similarly, those reporting an unmet mental health care need in the past year had a significantly higher prevalence of sleep apnea (11.3%) than those not reporting an unmet need (5.8%) and respondents reporting asthma in the past year (17.2%) than among those without such a report (5.4%).

**Table 2:** Prevalence of Sleep Apnea and Odds of Having the Condition Among US Male Veteran Respondents Aged ≥18 Years, by Selected Sociodemographic, Health, and Behavioral Characteristics, National Survey on Drug Use and Health, 2005–2014

Characteristic	Prevalence, % (95% CI)	*P* Value^a^	Adjusted OR (95% CI)	*P* Value^b^
**Age, y**				
18–34	2.6 (1.8–3.4)	<.001	1 [Reference]	
35–49	5.5 (4.6–6.4)		2.01 (1.40–2.88)	<.001
50–64	7.9 (6.9–8.8)		2.89 (2.02–4.12)	<.001
≥65	5.2 (4.5–5.8)		1.82 (1.23–2.70)	.003
**Race/ethnicity**				
Non-Hispanic white	4.1 (2.5–5.7)	.08	1 [Reference]	
Hispanic	6.6 (4.8–8.6)		0.71 (0.47–1.09)	.12
Non-Hispanic black	6.0 (5.5–6.5)		1.15 (0.82–1.62)	.41
Other	3.7 (1.9–5.5)		0.56 (0.33–0.97)	.04
**Marital status**				
Currently married	6.6 (6.0–7.1)	<.001	1 [Reference]	
Not currently married	4.4 (3.7–5.1)		0.66 (0.53–0.82)	<.001
**Federal poverty level**				
>200%	6.1 (5.6–6.6)	.055	1 [Reference]	
100%–200%	4.9 (4.0–5.7)		0.85 (0.69–1.03)	.10
<100%	5.6 (4.0–7.2)		0.94 (0.67–1.32)	.72
**Education**				
College graduate	5.6 (4.8–6.6)	.02	1 [Reference]	
Some college	7.1 (6.0–8.1)		1.28 (1.00–1.64)	.048
High school diploma or less	5.3 (4.7–6.0)		1.01 (0.81–1.25)	.97
**Current health insurance coverage**				
No	1.9 (1.0–2.7)	<.001	0.33 (0.21–0.51)	<.001
Yes	6.2 (5.7–6.6)		1 [Reference]	
**Past-year illicit drug or alcohol dependency**				
No	5.9 (5.5–6.4)	.53	1 [Reference]	
Yes	5.3 (3.4–7.2)		0.72 (0.47–1.10)	.13
**Past-year psychological distress**				
None	5.4 (4.8–5.8)	<.001	1 [Reference]	
Mild to moderate	7.5 (5.6–9.5)		1.61 (1.18–2.20)	.003
Serious	11.4 (9.5–13.3)		2.38 (1.84–3.06)	<.001
**Past-year unmet mental health care need**				
No	5.8 (5.3–6.2)	<.001	1 [Reference]	
Yes	11.3 (7.7–15.0)		1.61 (1.05–2.45)	.03
**Past-year asthma**				
No	5.4 (5.0–5.9)	<.001	1 [Reference]	
Yes	17.2 (13.0–21.4)		3.56 (2.55–4.97)	<.001

Abbreviations: CI, confidence interval; OR, odds ratio.

a *P* value from survey-weighted χ^2^ analyses.

b *P* value from survey-weighted logistic regression analyses in a single combined model with all characteristics listed in the table, in addition to survey year.

We found similar trends in multivariable logistic regression analyses (Table 2). Odds of sleep apnea were higher among respondents aged 50 to 64 years (adjusted odds ratio [aOR] = 2.89) than among those aged 18 to 34 and among those with some college education (aOR = 1.28) than among college graduates. Compared with those without any past-year psychological distress, those with mild to moderate past-year psychological distress had 61% higher odds of sleep apnea in the past year and those with serious psychological distress had 138% higher odds. Those reporting an unmet past-year mental health care need had 61% higher odds of sleep apnea than those reporting no unmet need, and those with a past-year diagnosis of asthma had 256% higher odds than among those without.

## Discussion

Our study demonstrated the 10-year trend of sleep apnea among US male veterans, and the burden of psychological distress and unmet mental health care need on sleep apnea. Such results highlight the need for multidisciplinary action to address mental illness, access to preventive health services, and comorbid conditions among this vulnerable population.

Peer-based programs improved mental health outcomes among police who responded to the events of September 11, 2001 (9). Similar peer-support strategies could improve veterans’ mental health status, increase use of health care services, and ultimately decrease the prevalence of chronic conditions such as sleep apnea. Moreover, community-based resources (10), such as faith-based initiatives, have improved access to health care services. Comparable initiatives could be implemented to screen veterans for sleep problems and mental illness and offer treatment or preventive services.

Another major finding that warrants further discussion is the prevalence of past-year asthma and its association with sleep apnea. Although the military’s policy of not allowing the recruitment of men and women with asthma (11) may eventually help to decrease the number of veterans with asthma, men and women who are asymptomatic when they are recruited may develop asthma caused by environmental risk factors during deployment (12). Routine screening for asthma among all veterans is needed, regardless of whether the service member had asthma symptoms during recruitment.

This study has limitations: NSDUH data are subject to the various biases of self-report (eg, social desirability, recall). Because of the cross-sectional design of our study, we could not establish any causal relationships. We could not assess other risk factors of sleep apnea because of the lack of questions on these risk factors in the NSDUH. We also could not determine whether the rise in sleep apnea was due to increased awareness of the condition or other factors; thus, other factors, beyond those examined in our study, need further exploration. Nevertheless, the survey-weighted data allow for generalizability of the study results to US male veterans, and the 10-year trend provides additional insight into the burden of sleep apnea among US male veterans.

Our study is unique in that it demonstrates a putative relationship between sleep apnea and mental illness. Although mental health is a major public health issue, especially among US veterans, few studies have examined many of the comorbid conditions that may be associated with mental illness among this vulnerable population. Our study expands this research and highlights the need for more rigorous screening of sleep apnea and better sleep apnea treatment among US male veterans.
